# Crystal structure of *O*-methyltransferase CalO6 from the calicheamicin biosynthetic pathway: a case of challenging structure determination at low resolution

**DOI:** 10.1186/s12900-015-0040-6

**Published:** 2015-07-15

**Authors:** Oleg V. Tsodikov, Caixia Hou, Christopher T. Walsh, Sylvie Garneau-Tsodikova

**Affiliations:** Department of Pharmaceutical Sciences, College of Pharmacy, University of Kentucky, 789 South Limestone Street, 40536-0596 Lexington, KY USA; Department of Biological Chemistry and Molecular Pharmacology, Harvard Medical School, 200 Longwood Avenue, 02215 Boston, MA USA

**Keywords:** Anticancer drug, Enediyne, Low-resolution refinement, Methylation, Natural product biosynthesis

## Abstract

**Background:**

Calicheamicins (CAL) are enedyine natural products with potent antibiotic and cytotoxic activity, used in anticancer therapy. The *O*-methyltransferase CalO6 is proposed to catalyze methylation of the hydroxyl moiety at the C2 position of the orsellinic acid group of CAL.

**Results:**

Crystals of CalO6 diffracted non-isotropically, with the usable data extending to 3.4 Å. While no single method of crystal structure determination yielded a structure of CalO6, we were able to determine its structure by using molecular replacement-guided single wavelength anomalous dispersion by using diffraction data from native crystals of CalO6 and a highly non-isomorphous mercury derivative. The structure of CalO6 reveals the methyltransferase fold and dimeric organization characteristic of small molecule *O*-methyltransferases involved in secondary metabolism in bacteria and plants. Uncommonly, CalO6 was crystallized in the absence of *S*-adenosylmethionine (SAM; the methyl donor) or *S*-adenosylhomocysteine (SAH; its product).

**Conclusions:**

Likely as a consequence of the dynamic nature of CalO6 in the absence of its cofactor, the central region of CalO6, which forms a helical lid-like structure near the active site in CalO6 and similar enzymes, is not observed in the electron density. We propose that this region controls the entry of SAM into and the exit of SAH from the active site of CalO6 and shapes the active site for substrate binding and catalysis.

**Electronic supplementary material:**

The online version of this article (doi:10.1186/s12900-015-0040-6) contains supplementary material, which is available to authorized users.

## Background

**Fig. 1:**
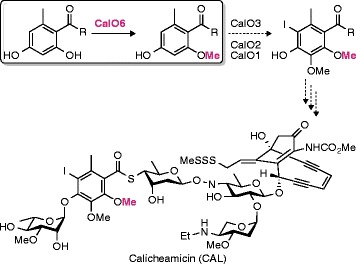
Proposed *O*-methylation of orsellinic acid likely tethered to the ACP domain of CalO5 (indicated by an R) by CalO6 during calicheamicin (CAL) biosynthesis

Calicheamicins (CAL) are bacterial natural products with antibiotic and antitumor activities, which originate from the ability of these compounds to cleave double-stranded DNA [1]. CAL, previously considered too toxic to be used in clinic, is now being reevaluated as an antibody-conjugated therapeutic used in combination with other anticancer drugs in treatment of acute leukemias [2, 3]. The structure of CAL is composed of a reactive aglycone responsible for double-stranded DNA breaks, and a sugar-rich tail containing a thiobenzoate bridging group, orsellinic acid, elaborated with two methoxy groups at positions C2 and C3, a 6-methyl group, and a 5-iodine moiety (Fig. 1). This thiobenzoate moiety was shown to be a determinant of the DNA sequence specificity of CAL [4–6]. Protein CalO6 from the CAL gene cluster was originally proposed to catalyze one or both *O*-methylations (at positions C2 and/or C3) of this moiety [7]. A recent report suggests that the 2-hydroxyl group methylation is carried out by CalO6 as the first tailoring reaction of the orsellinic acid moiety likely attached to the acyl carrier protein (ACP) domain of CalO5 (indicated by R in Fig. 1) [8], whereas another candidate *O*-methyltransferase, CalO1, is inert when tested on the same substrate analogue [9].

Homologues of CalO6 are dimeric SAM-dependent *O*-methyltransferases from bacteria and plants acting predominantly on hydroxyl groups of aromatic compounds involved in secondary metabolism. The founding member of this family is DnrK, an *O*-methyltransferase involved in the biosynthesis of the anticancer agent daunorubicin [10]. Methyltransferases of this family consist of three domains: the N-terminal helical domain is responsible for the dimerization via domain swapping, the central helical domain forms a lid over the active site, and the C-terminal Rossmann-fold domain bears a SAM binding motif and the catalytic residues, and forms a part of the substrate-binding pocket. Despite 30-40 % sequence identity among the methyltransferases of this family, structures of these enzymes exhibit numerous differences in mutual disposition of the domains, secondary structural elements, and other structural variability, likely as a result of evolutionary divergence to accept substrates of different structures and sizes. In many cases, including CalO6, endogenous substrates of these enzymes are not known, as their determination depends on the detailed elucidation of the biosynthetic pathways. Therefore, structural information can help shed light into these complex assembly processes. In this study, we determine a crystal structure of the *O*-methyltransferase CalO6.

## Methods

### Materials and instrumentation

Chemically competent *E. coli* TOP10 and BL21(DE3) cells were purchased from Invitrogen. *Pfu* DNA polymerase was from Stratagene. T4 DNA ligase was from New England BioLabs. DNA primers were from Integrated DNA Technologies. The pET28a was from Novagen. Cell disruption was performed with a QSonica Q500 sonicator. Gel filtration was performed on a fast protein liquid chromatography (FPLC) Bio-Rad BioLogic DuoFlow system using a HighPrep™ 20/60 Sephacryl™ S-200 HR column. Amicon Ultra-15 filtration unit was from Millipore.

### Construction of the pCalO6-pET28a overexpression clone

The *calO6* gene was amplified by polymerase chain reaction (PCR) from *Micromonospora echinospora* genomic DNA with *Pfu* DNA polymerase using the forward primer 5′-GTCATACATATGGAACTCACCACGACCG-3′ and the reverse primer 5′-CAGTGCCTCGAGTCAGCTCCCGTCCGG-3′, which introduced a *Nde*I and a *Xho*I restriction site (underlined), respectively. The resulting PCR fragment was inserted into the *Nde*I and *Xho*I sites of the linearized *E. coli* expression vector pET28a, generating a construct of CalO6 bearing an N-terminal hexa-histidine tag cleavable by thrombin. After transformation into chemically competent *E. coli* TOP10 cells, the pCalO6-pET28a DNA was isolated. The sequencing of the construct yielded an insert sequence that was in perfect agreement with the annotated sequence of *calO6* from *Micromonospora echinospora* (accession number: AAM70356).

### Expression and purification of CalO6

The pCalO6-pET28a construct was transformed into chemically competent *E. coli* BL21(DE3) cells. A 1 % inoculum of transformants containing the pCalO6-pET28a was grown (in 6 × 1 L; 37 °C, shaking at 200 rpm) in Luria-Bertani (LB) broth supplemented with kanamycin (50 μg/mL) until attenuance of 0.6 at 600 nm. After induction with 100 μM isopropyl-1-thio-β-galactopyranoside (IPTG), the cultures were grown for 17 h at 25 °C. Cells were harvested by centrifugation at 5,000 × g for 15 min at 4 °C, and resuspended in lysis buffer [25 mM Tris-HCl (pH 8.0, adjusted at room temperature (rt)), 400 mM NaCl, and 10 % (v/v) glycerol]. After cell disruption by intermittent sonication on ice and removal of the cell debris by centrifugation at 40,000 × g for 45 min at 4 °C, the clarified lysate was passed through a 0.45 μm PVDF filter, and then imidazole was added at a final concentration of 2 mM. The lysate was loaded onto a Ni-affinity chromatography column (5 mL HP HiTrap IMAC column; GE Healthcare), followed by 3 × 5 mL of lysis buffer with 40 mM imidazole, 3 × 5 mL of lysis buffer with 100 mM imidazole, and 6 × 5 mL of lysis buffer with 200 mM imidazole. Fractions containing pure CalO6, as determined by SDS-PAGE, were pooled and dialyzed overnight in dialysis buffer [50 mM Tris-HCl pH 8.0, 100 mM NaCl, 0.1 mM EDTA (pH 8.0, adjusted at room temperature), and 10 % (v/v) glycerol]. The protein was concentrated to 3 mg/mL, and the hexa-histidine tag was cut at 4 °C for 30 h by thrombin. CalO6 was then purified away from the tag and thrombin by gel filtration (GF) chromatography in GF buffer [50 mM Tris-HCl (pH 8.0, adjusted at rt), 100 mM NaCl, 0.1 mM EDTA, and 1 mM DTT]. Fractions containing pure CalO6 (as determined by SDS-PAGE), were pooled and the protein was concentrated to ~14 mg/mL in an Amicon Ultra-15 filtration unit and stored at 4 °C for use in crystallization experiments. The purified protein (Additional file 1: Figure S1 in Supporting information) was assayed as reported previously [8] and exhibited similar activity in methylating *S*-*N*-acetylcysteaminyl orsellinic acid, as detected by HPLC. SeMet-substituted CalO6 was prepared as previously described [11] and purified analogously to the unsubstituted CalO6.

### Crystallization, data collection, and crystal structure determination

Crystals of CalO6 were grown by vapor diffusion in hanging drops made by mixing 1 μL of protein with 1 μL of crystallization buffer [0.1 M NaCl, 0.1 M Bis-Tris (pH 5.8, adjusted with HCl at room temperature), and 1.10-1.25 M ammonium sulfate], incubated over 1 mL of crystallization buffer. Rod shaped crystals of CalO6 (0.1 mm × 0.1 mm × 0.3 mm in size) grew in 3 days at 21 °C and were transferred to cryoprotectant solution [0.1 M NaCl, 0.1 M Bis-Tris pH 5.8 (adjusted with HCl at room temperature), 1.10-1.25 M ammonium sulfate, 15 % (v/v) glycerol] by increasing glycerol concentration in steps of 3 %, then incubated there for 40 min and quickly immersed into liquid nitrogen. SeMet-substituted CalO6 displayed much lower solubility (precipitated at ~3 mg/mL) than unsubstituted CalO6 due to a large number of Met residues. Crystals of SeMet CalO6 took 2-3 weeks to grow and did not diffract well enough (resolution >4 Å, streaky reflections) to be useful for phasing. Likewise, CalO6 did not form suitable crystals in the presence of 1-2 mM SAM, SAH, or 2-5 mM substrate *N*-acetylcysteamine orsellinic acid (SNAC-OSA; used with or without SAH), nor did we observe these ligands in the electron density map upon soaking them into crystals of CalO6 grown in their absence. These ligand concentrations were several-fold higher than the previously reported *K*_m_ values (0.3 mM for SAM and 1.3 mM for SNAC-OSA) [8], which ensured that most CalO6 is in a ligand-bound form at the conditions of the reported activity assays. However, we could not exclude a possibility that the ligand binding was disfavored in the crystallization solution. Ethyl mercury phosphate (EMP)-derivative crystals of CalO6 were prepared by soaking crystals of native CalO6 in the cryoprotectant solution containing 2 mM of EMP overnight prior to flash-freezing in liquid nitrogen. A number of other mercury, platinum, tantalum, and other reactive and inert heavy metal salts were tried, but did not yield useful derivatives.

X-ray diffraction data were collected at beamline X-12 at the National Synchrotron Light Source at the Brookhaven National Laboratory and processed with HKL2000 [12]. The diffraction was highly anisotropic, with the useful data extending only to a modest-low resolution (Table 1), making structure determination challenging. The anisotropy analysis by the anisotropy server [13] indicated that the data were strongly anisotropic (the spread in values of the three principal components of scale factors is 33.62 Å^2^), with resolution limits of 3.6 Å, 3.6 Å, and 3.1 Å along three principal component axes. The crystals were not merohedrally twinned, as analyzed by using XTRIAGE [14] program in PHENIX suite [15]. In addition, EMP derivative crystals were highly non-isomorphous with native CalO6 crystals. Three-wavelength data set was collected with the EMP derivative, but due to rapid crystal decay in the X-ray beam, only the data set collected at 1.007 Å was used for structure determination. The anomalous signal was measurable to 4.1 Å according to XTRIAGE output; with 25 % of strong (>3σ) intensities displaying strong anomalous signal (the magnitude of the Bijvoet intensity difference over 3σ) in the lowest resolution shell and 5 % of strong intensities with strong anomalous signal at resolution ~4.1 Å. Four mercury sites were found by SOLVE [16], but the resulting electron density map quality was insufficient for model building.

**Table 1:** X-ray diffraction data collection and refinement statistics for CalO6

Data collection	EMP^a^ derivative	Native
Space group	R32	R32
Number of monomers per asymmetric unit	1	1
Unit cell dimensions		
a, b, c (Å)	126.8, 126.8, 105.7	130.0, 130.0, 105.2
α, β, γ (°)	90, 90, 120	90, 90, 120
Resolution (Å)	50.0-3.1 (3.2-3.1)^b^	50.0-3.1 (3.15-3.10)
I/σ	28 (1.9)	34.3 (2.6)
Completeness (%)	98.1 (92.8)	99.7 (98.3)
Redundancy	3.4 (3.2)	7.1 (5.4)
R_meas_	0.046 (0.571)	0.077 (0.697)
C_1/2_^c^ in the highest resolution shell	0.76	0.89
Number of unique reflections	11759 (1102)	6322 (296)
Structure refinement statistics		
Resolution (Å)^d^	25.0-3.4	
R (%)	32.5	
R_free_ (%)	33.3	
Number of non-hydrogen atoms	2194	
Bond length deviation (rmsd) from ideal (Å)	0.009	
Bond angle deviation (rmsd) from ideal (°)	1.44	
Clashscore	6	
Ramachandran plot statistics^e^		
% residues in allowed regions	93.7	
% residues in generously allowed regions	3.9	
% residues in disallowed regions	2.4	

^a^EMP stands for ethyl mercury phosphate

^b^Numbers in parentheses indicate the values in the highest-resolution shell

^c^C_1/2_ is calculated as defined previously [31]

^d^Due to strong anisotropy, data to 3.4 Å was usable in the refinement

^e^Indicates Procheck [32] statistics

**Fig. 2:**
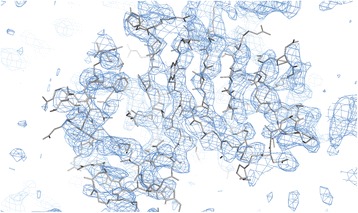
A fragment of the electron density map after density modification in AUTOSOLVE, contoured at 1 rmsd. The respective part of the refined CalO6 structure is shown in grey sticks as a reference

Molecular replacement (MR) was attempted by using the data collected with the native CalO6 crystal with all available crystal structures of different CalO6 homologues as search models, by using PHASER [17] and MOLREP [18]. The only structure that yielded a molecular replacement (MR) solution was that of aclacinomycin-10-hydroxylase RdmB (PDB ID: 1QZZ) [19], the only known non-methyltransferase in this structural family. Specifically, only the C-terminal domain of RdmB as a search model yielded an MR solution; neither searching with the full-length RdmB nor searching with the N-terminal domain of RdmB after placing the C-terminal domain were productive. The resulting electron density map was not of high enough quality for model building. However, the phase provided by the MR solution when used with the anomalous difference signal from the EMP derivative SAD data yielded 2 mercury sites in the anomalous difference Fourier map. With these mercury sites as an input, we used AUTOSOLVE [20, 21] in PHENIX package [15], to combine the MR and the SAD phases to find 5 additional sites and yield an interpretable electron density map with the figure of merit of 0.46 after density modification (Fig. 2). The electron density for the missing N-terminal domain was clearly discernible in the difference density map. The structure was then built by ~50 cycles of iterative model building with Coot [22] and refinement with REFMAC [23] by using the EMP derivative data set. Tight geometric restraints were used in REFMAC to prevent divergence and preserve proper bond geometry, which also kept *R* and *R*_free_ values similar to each other. The resolution cut-off was chosen as 3.4 Å, as no map or statistic improvement was achieved upon including higher resolution data in the refinement, also consistent with the anisotropy analysis. Using the data extending to 3.1 Å in resolution after the ellipsoidal truncation by the anisotropy server did not lead to improvement either. Potential twinning in R3 space group to mimic apparent R32 was excluded based on the Britton plot analysis by XTRIAGE program [15]. Refinement of a model that contained two CalO6 monomers per asymmetric unit with the data reduced in R3 did not yield further improvement in map quality or refinement statistics. All mercury sites were located near sulfur atoms of the Cys residues of the refined structure, confirming the proper residue register. The data collection and refinement statistics are given in Table 1. Due to an apparently complex non-isomorphism and very high anisotropy, the native CalO6 crystal data did not improve the resolution or map quality, even after molecular replacement with individual domains as search models.

## Results and discussion

CalO6 is a rare *O*-methyltransferase crystallized in the absence of cofactors or substrates; in fact co-crystals with its ligands either did not grow (with SAH) or were not of high enough quality for data collection (with SAM), and crystal soaking experiments did not yield stably bound complexes, likely because those were disfavored by the crystallization conditions or crystal packing. The different behavior of CalO6 in the absence and presence of SAM and SAH in the crystallization experiments suggested that the protein may undergo conformational changes upon cofactor binding. The crystal structure of CalO6 was determined by a combination of molecular replacement by using diffraction data collected with native crystals with single anomalous dispersion (SAD) using an EMP derivative, as described in Materials and Methods. Locations of all mercury sites were consistent with covalent Cys modification. These were only partially occupied, explaining insufficient phasing power of the SAD data alone. The partial occupancy may have been caused by poor steric accessibility of Cys thiols or quenching of EMP by nucleophilic ammonia generated by high concentrations of ammonium sulfate [24]. However, because of severe anisotropy of the native crystal data and strong non-isomorphism between the native and the mercury derivative data, only the EMP derivative data set was used throughout the structure building and refinement process.

**Fig. 3:**
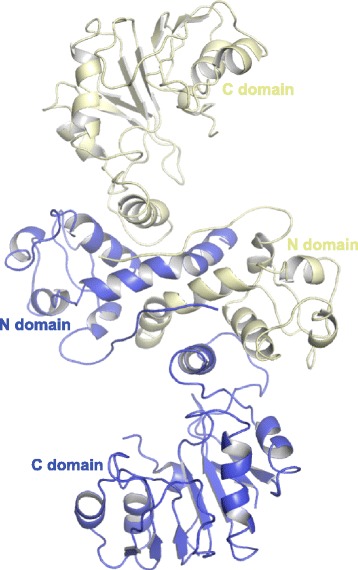
A cartoon representation of the structure of CalO6 dimer. The second monomer was generated by a crystal symmetry operation

**Fig. 4:**
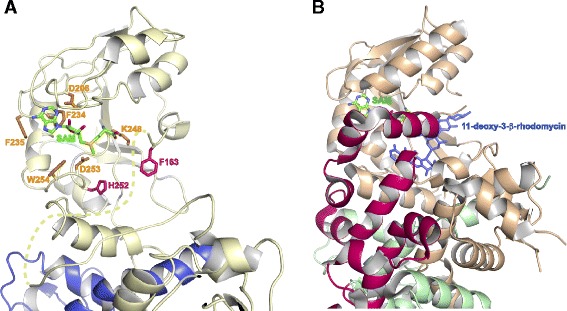
**a**. The active site of CalO6. A SAM molecule (colored sticks with C in green, N in blue, O in red, and S in yellow) was modeled to be bound to CalO6 similarly to its binding to RdmB (PDB ID: 1XDS [28]) with confidence based on highly superimposable SAM binding motifs of CalO6 and RdmB. The disordered middle domain is shown by the dashed curve. The SAM interacting residues are shown in orange sticks; the proposed catalytic His252 and a putative substrate binding Phe163 are shown as red sticks. **b**. Structure of the active site of RdmB in complex with SAM (same colors as panel **a**) and 11-deoxy-3-β-rhodomycin (blue sticks). The folded middle domain capping the active site is shown in red

Crystals of CalO6 belong to space group R32, with one monomer in the asymmetric unit (Table 1). The other monomer in the dimer is generated by a crystal symmetry operation (Fig. 3). Methyltransferases similar to CalO6 in sequence and structure, with sequence identity to CalO6 in the 30-40 % range, occur in all three domains of life; they perform *O*-methylation in biosynthesis of secondary metabolites and signaling molecules. Examples of such methyltransferases include caffeic acid *O*-methyltransferase from perennial ryegrass [25], human *N*-acetyl serotonin *O*-methyltransferase [26], as well as chalcone and isoflavone *O*-methyltransferases from alphalpha [27]. It is proposed that the catalysis in these enzymes occurs through the activation of the hydroxyl group to be methylated through abstracting its hydrogen by a catalytic His residue in the enzyme active site (His252 in CalO6; Fig. 4a). This phenolate group then acts as a nucleophile and attacks the electrophilic methyl carbon of SAM. The list of similar proteins also includes SAM-dependent aclacinomycin-10-hydroxylase RdmB, in which the active site His residue is replaced by a Leu, and there is no other residue that could act as a catalytic base within 7 Å of the methyl group of SAM [19]. Instead of methylation, RdmB catalyzes decarboxylation (the resulting carbanion is stabilized by SAM) followed by oxidation through formation of a hydroxyperoxide intermediate [28]. This example illustrates divergence of not only the substrates, but also the catalytic functions in this enzyme family. Similarly to these homologues, CalO6 consists of three domains. The entirely helical N-terminal domain of CalO6 (residues 1 to 105) is involved in dimerization, and the C-terminal Rossman-fold domain (residues 162 to 356) containing the SAM-binding motif, is involved in substrate binding and the catalysis of the transfer of the methyl group from SAM onto a hydroxyl group of the substrate. A normally helical region between these two domains (residues 106 to 161; called a middle domain in some studies), which forms a part of the substrate-binding pocket in other methyltransferases, is disordered and not visible in the electron density map (as indicated by a dashed line in Fig. 4a). This region appears to act as a lid that is closed onto a bound substrate, as exemplified by the structure of RdmB in complex with SAM and 11-deoxy-3-β-rhodomycin (Fig. 4b) [28]. As a consequence, the substrate-binding pocket in CalO6 is much more open than in other *O*-methyltransferase structures. Disorder in this region has been observed previously in homologues of CalO6, including chalcone *O*-methyltransferase [27] and RdmB [28]. Furthermore, a recent series of crystal structures of a more distant single-domain homologue outside of the DnrK family, human catechol-*O*-methyltransferase, an important drug target of nervous system disorders, display significant disorder in respective regions (termed α2/α3 in that system) in the apo-form, with ordering and closing these region onto the active site upon binding of SAM and/or inhibitors that mimic SAM or the substrate [29]. Therefore, disorder of this region in the absence of bound substrate or co-substrate appears to be common among this broad class of *O*-methyltransferases. CalO6 appears to be the most extreme case, where the entire middle region is disordered or is in different positions relative to the rest of the protein in different CalO6 monomers in the crystal. Because a part of the middle domain interacts with SAM in other similar methyltransferases, this region likely undergoes at least partial coupled folding upon SAM binding. The less ordered state of CalO6, crystallized in the apo-form, explains why crystal structures of homologues of this methyltransferase in the apo form are extremely rare, since disorder or dynamic nature is associated with poor crystallizability [30].

The N-terminal domain is oriented at a different angle with respect to the catalytic domain from that seen in other methyltransferases bound to SAM, opening the active site even further. For example, with the C-terminal domains superimposed, the tip of the N-terminal domain furthest from the pivot point is located 13 Å away from its position in RdmB (the closest CalO6 structural homologue with the rmsd of the distances between the Cα atoms of 1.5 Å for the C-terminal domain) and 15 Å away from its position in mitomycin *C*-methyltransferase MmcR (the closest CalO6 sequence homologue). These differences correspond to rotations of the N-terminal domains by ~35° for RdmB and MmcR. Because the N-terminal domain interacts with the middle domain in the ligand-bound structures of similar enzymes (Fig. 4b), the orientation of the N-terminal domain and the conformation of the middle domain likely change in a concerted way upon binding of the co-substrate and the substrate, in an induced-fit mechanism. The difference in the relative orientation of the N-terminal and the C-terminal domains for the same methyltransferase depending on a bound substrate was previously observed in RdmB [28] and recognized as the conserved feature allowing the adaptability of the same structural fold to a wide variety of substrates. These observations are also consistent with the different crystallization properties of ligand bound and apo CalO6, and strongly suggest that CalO6 and similar enzymes undergo a conformational change upon their co-substrate and substrate binding.

## Conclusions

An example of challenging structure determination, CalO6, a dimeric Dnrk family *O*-methyltransferase, was crystallized in the absence of a cofactor or a substrate. The structure of CalO6 indicates a dynamic nature of the middle domain, which serves as an active site lid in this unbound form, as well as suggests that the relative disposition of all the domains changes to position the co-substrate and the substrate for catalysis.

### Availability of supporting data

The structure factor amplitudes and atomic coordinates are available in the Protein Data Bank repository, Accession Code 4Z2Y.

## Additional file

Additional file 1:
**The supporting information contains a figure showing the coomasie blue-stained 15 % Tris-HCl SDS-PAGE gel of the purified CalO6 protein used for crystallization studies.**

## Abbreviations

ACP: Acyl carrier protein
CAL: Calicheamicin
EMP: Ethyl mercury phosphate
FPLC: Fast protein liquid chromatography
GF: Gel filtration
IPTG: Isopropyl-1-thio-β-galactopyranoside
LB: Luria-Bertani
MR: Molecular replacement
PCR: Polymerase chain reaction
SAH: *S*-adenosylhomocysteine
SAM: *S*-adenosylmethionine
SNAC-OSA: *N*-acetylcysteamine orsellinic acid
