# Supplementary material for: Crystal structure of O-methyltransferase CalO6 from the calicheamicin biosynthetic pathway: a case of challenging structure determination at low resolution
Source: BMC Struct Biol. 2015 Jul 15;15:13. doi: 10.1186/s12900-015-0040-6 (PMC4501126; doi:10.1186/s12900-015-0040-6)
Supplement: Additional file 1: — The supporting information contains a figure showing the coomasie blue-stained 15 % Tris-HCl SDS-PAGE gel of the purified CalO6 protein used for crystallization studies. [file 12900_2015_40_MOESM1_ESM.pdf]

SUPPORTING INFORMATION

Crystal structure of *O*-methyltransferase CalO6 from the calicheamicin biosynthetic pathway: a case of challenging structure determination at low resolution

Oleg V. Tsodikov,[a,*] Caixia Hou,[a] Christopher T. Walsh,[b] and Sylvie Garneau-Tsodikova[a,*]

[a] Department of Pharmaceutical Sciences, College of Pharmacy, University of Kentucky, 789 South Limestone Street, Lexington, KY, 40536-0596, USA
[b] Department of Biological Chemistry and Molecular Pharmacology, Harvard Medical School, 200 Longwood Avenue, Boston, MA, 02215, USA

*Correspondence e-mails: oleg.tsodikov@uky.edu
sylviegtsodikova@uky.edu

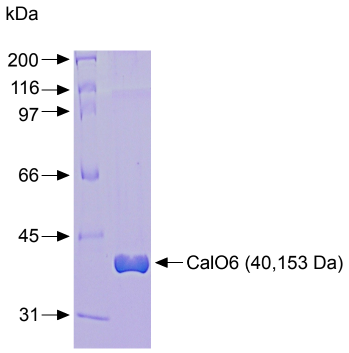

Fig. S1. Coomasie blue-stained 15% Tris-HCl SDS-PAGE gel showing the pure CalO6 protein used for crystallization studies. 6 μg of protein was loaded on the gel. The molecular weight ladder used was SDS-PAGE Molecular Weight Standards, Broad Range (Bio-Rad cat# 161-0317).
